# Physical fitness and levels of physical activity in people with severe mental illness: a cross-sectional study

**DOI:** 10.1186/s13102-017-0082-0

**Published:** 2017-11-25

**Authors:** David Perez-Cruzado, Antonio I. Cuesta-Vargas, Elisa Vera-Garcia, Fermín Mayoral-Cleries

**Affiliations:** 1 Department of Physiotherapy, Faculty of Helath Sciences, University of Malaga, Av/ Arquitecto Peñalosa s/n, Malaga, Spain; 2 School of Clinical Science, Faculty of Health Science, Queensland University Technology, Brisbane, QLD Australia; 3 Mental Health Research Group of Biomedicine Research Institute of Malaga (IBIMA) Spain, Regional University Hospital, Malaga, Spain; 4 Clinimetric Research Group of Biomedicine Research Institute of Malaga (IBIMA) Spain, Regional University Hospital, Málaga, Spain

**Keywords:** Schizophrenia, Physical illness, Physical activity, Physical fitness, Psychiatry

## Abstract

**Background:**

Physical fitness is a crucial variable in people with severe mental illness as these people could be more independent and improve their job opportunities. The present study compared the physical fitness of physically active and inactive people with severe mental illness.

**Methods:**

Physical fitness was evaluated in sixty-two people with severe mental illness using 11 physical tests that include strength, flexibility, balance and aerobic condition. Significant differences were found between both groups in muscle strength (handgrip test) and balance (single leg balance test and functional reach) with better performance in the group of physically active people.

**Results:**

The results of the present study suggest that physical fitness (strength and balance) is higher in people with severe mental illness who practise regular physical activity that those who are inactive people.

**Conclusions:**

Physical active people may have a reduced risk of falls and fractures due to their higher levels of physical fitness.

## Background

Severe mental illness (SMI) applies to all those people with functional psychosis over 18 who have an ICD-10 diagnosis of an affective or non-affective functional psychotic disorder (Codes F10–F22, F24, F25, F28–F31. F32.3, F33.3) [1]. People with SMI have a decrease of at least 20% in life expectancy due to premature death and the increase of cardiovascular disease [2, 3]. High levels of risk of death in this population reflects a combination of factors, such as the increased prevalence of different diseases, adverse effects of drug treatment or poor access to physical care and physical activity [4].

Physical activity provides significant health benefits to people with SMI, reducing the risk of many diseases of this population [5–7], representing an increase in job opportunities and greater independence in performing activities of daily living [8, 9], so that the participation of adults with severe mental illness in sports and recreational activities has often been addressed to enhance overall wellbeing and promote social inclusion [10–12].

Despite all the benefits provided by physical activity for people with SMI these people find many barriers to this physical activity leading them to lack motivation and self-efficacy for independent physical activity [13]. For instance, there is a lack of social support by professionals and family to practise physical activity and a lack of physical activity priority over other mental health treatments [14, 15], so this population is a group at risk of all negative consequences of a sedentary lifestyle [16, 17].

The aim of the present study is to determine the levels of physical fitness of people with SMI and identify any differences between their level of physical fitness and amount of physical activity.

## Methods

### Participants

In the present study, 62 people with SMI (37 men and 25 women) participated. They were aged between 26 and 61 years, and recruited from the Hospital Civil, Malaga (Spain) between March and May of 2015. All participants were inpatients and had been diagnosed with a pathology included in SMI. Their general health was checked with a physical examination prior to participation of these people in the study. Participants included in the present study had not experienced exacerbation of their symptoms and were not suffering from any disease that limited them in physical activity or physical tests. Participants attended the Hospital Civil 4 h a day from Monday to Friday. The participants had all the rest of the day free to participate in activities that involve the practice of physical activity. The participants were independent people when deciding whether or not they wanted to perform physical activity.

Participants were asked about their adherence to physical activity, and the hours per week that they practised physical activity (30 min per day, moderate intensity) were recorded to divide the sample according to their physical activity level. Data about self-reported physical activity was ensured by staff and family members, since the information provided by the participants was contrasted by the information given by the relatives and the staff. There were no discrepancies between the information given by the participants and that granted by the workers and their families. Participants were identified as physically active or inactive according to physical activity engaged 3–7 days per week (active) or 1–2 days per week (inactive) [18].

### Study design and procedure

A cross-sectional study was performed. Two examiners assessed the physical fitness in people with SMI with 11 physical tests to assess flexibility, strength, balance and aerobic condition. Physical tests were explained to participants and the examiners demonstrated how the test should be performed if they did not understand.

#### Physical tests were

- Passive knee extension (PKE): Each participant is positioned supine on a stretcher with hip and knee flexed at 90 degrees. This test is evaluated with the aid of a goniometer. If the knee attains full extension, it is recorded as 0°, whereas if the extension fails to register, it has a negative value [19]. The reliability of the PKE test in this population is excellent (0.95–0.98) [20].
- Calf muscle flexibility (CMF): The participant is placed supine on a stretcher, with hip and knee at the maximum extent possible. Each participant is asked to perform a dorsiflexion of the foot. If the participant cannot get to that position, the angle is recorded as negative, whereas if it goes beyond the neutral position, it is recorded as positive [21]. The reliability of this test in people with ID can be found in Waninge et al. (0.86–0.93) [22].
- Anterior hip flexibility (AHF): The participant is placed supine with both hips flexed at 90°. Measured hip is flexed to 100° with one hand under the lower back to make sure it does not rise. Degrees of extension between the pelvis and thigh before the pelvis begins to move forward are measured. If the thigh extends down to the table, this is recorded as 0°, and if it does not extend to the table, the angle is recorded as negative [21]. Validity of this test can be found in “Brockport Physical Fitness Test Manual: A Health-Related Assessment for Youths with Physical and Mental Disabilities” [23].
- Functional shoulder rotation (FSR): The participant is placed standing and should position the arm behind the head and back and the other arm behind the hip and back. The participant should try touching their index fingers, and the distance is measured with a tape. The distance is negative if they fail to touch their fingers and positive if contact is made. The upper arm defines the side to evaluate [24]. The reliability of this test can be found in Edwards et al. (2002) [25].
- The time-stands test (TST): This test is used to evaluate the strength of the lower extremities. The participant must sit upon and rise from the chair 10 times, as fast as possible without using their arms. The time it takes the participant to complete the exercise is recorded [26]. The reliability of this test can be found in Newcomer et al. (1993) [26].
- Partial sit-up test (PSUT): This test is used to measure abdominal strength. The participant must make the maximum possible number of abdominal contractions in a minute from a supine position with their legs placed on a chair or weight to maintain knee and hips flexed at 90° [27]. Test-retest reliability and validity was established in a previous study [28].
- Seated push-up (SPU): This test is used to evaluate the strength off triceps, shoulders and pectoral muscle. In this test, each participant must raise their body from a sitting position (with extended knees) until elbows are straight for 30 s. The length of time they remain in this position is recorded [29]. Reliability and validity of the test are shown in the study of Graham & Reid [30].
- Handgrip test (HGT): This is a standardized method to assess the strength of the muscles of the hand and forearm. The arm must remain with the elbow bent at 90 degrees [31]. Reliability and validity of the test are shown in the study of Graham & Reid [30].
- Single leg stance (SLS): This test is used to evaluate static balance. The participant must stand on one leg for as long as possible. The second time that each participant remains in this position is recorded (maximum 60 s). The arms must remain on the hip with elbows slightly bent. The test is performed with each supported leg and with eyes open (EO) [32] and eyes closed (EC) [33]. Validity and reliability of both test can be found in the study of Lahtinen et al. [28].
- Functional reach test (FRT): In this test, the participant must reach as far as possible without loss of balance. For this, the participants stand with feet should width apart, with arms bent at 90 degrees and extended fingers. In this position, participants lean forward and the maximum length reached is recorded in centimetres [34]. Test-retest reliability was established in the study of Cuesta-Vargas & Gine-Garriga [35].
- Two-minute step test (2MST): In this test, the participant is placed near a wall where the minimum height is marked to where legs must be raised. Each participant must perform a step-up in place for 2 minutes, lifting her legs to the level that appears on the wall. Heart rate is evaluated at rest, just after completion of the test and 2 minutes after finishing the test [36]. This test have been validated in people disabilities [37].

### Ethical issues

The study was approval approved by the Ethics Committee of the University of Malaga and was carried out following the principles of the Declaration of Helsinki. At all times the anonymity of the participants was guaranteed according to the Data Protection Law.

Prior to participate in the study, an informed consent and a document with frequently asked questions were read by the participants. A written consent was obtained of all participants of the study.

### Statistical analysis

Descriptive data for all variables were presented as mean and standard deviation. The normal distribution of data was assessed with the Kolmogorox-Smirnov test. Depending on the results of the test, a t-test or Wilconxon test was applied. Data analysis was performed with SPSS version 22.0 statistical software.

## Results

In the present study, 62 people with SMI were evaluated and all participants completed all the physical tests with no missing data. No participants withdrew from the study or refused to perform any physical test. Of the 62 participants, 21 (33.87%) were categorized as physically active because they engage in physical activity 3–7 days per week, while 41 participants (66.13%) were categorized as physically inactive. The average height of the sample was 164.77 (±20.81) centimetres, and the average weight was 81.25 (15.84) kilogrammes, with a waist circumference of 103.04 (±13.53) centimetres and a BMI (body mass index) of 29.86 (±3.12), with no significant differences between the active and inactive participants. The average age of the sample was 46.21 (±8.37) years with no significant differences neither.

Descriptive and inferential data about physical fitness are presented in Table 1. Significant differences were found between physically active group and physically inactive group in one strength test (handgrip test) and in balances tests (SLS with closed eyes and FRT with left leg.

**Table 1:** T-student differences of the physical fitness test between both groups

		Physically active (mean ± sd)	Physically inactive (mean ± sd)	Difference (student-t)
Flexibility	Passive knee extension _right (°)	−26.59 (±16.52)	−24.18 (±13.84)	0.39
	Passive knee extension _left (°)	−22.95 (±17.79)	−25.62 (±16.00)	0.72
	Calf muscle flexibility _right (°)	−0.95 (±3.19)	−0.73 (±1.91)	−0.27
	Calf muscle flexibility _left (°)	−1.95 (±5.52)	−1.38 (±2.39)	−0.34
	Anterior hip flexibility _right (°)	−0.44 (±2.08)	0.03 (±1.13)	−0.92
	Anterior hip flexibility _left (°)	−0.44 (±2.08)	0.02 (±0.98)	−0.92
	Functional shoulder rotation _right (°)	−14.18 (±15.39)	−11.62 (±14.00)	−0.18
	Functional shoulder rotation _left (°)	−20.15 (±14.5)	−15.46 (±14.68)	−0.44
Strenght	Time-stands test (s)	25.94 (±14.88)	26.69 (±11.01)	−0.81
	Partial sit-up test (Repetition/1 m)	25.79 (±12.47)	26.20 (±8.87)	−0.34
	Seated push-up (s)	15.11 (±7.60)	15.26 (±10.80)	−0.57
	Handgrip test _right (kg)	27.78 (±11.98)	29.18 (±13.57)	−0.22*
	Handgrip test _left (kg)	24.44 (±11.84)	26.83 (± 11.09)	0.53*
Balance	Single-leg stance _OE_right (s)	14.39 (±11.97)	15.68 (±12.09)	0.18
	Single-leg stance _OE_left (s)	10.58 (±9.41)	16.73 (±11.47)	−2.04
	Single-leg stance _CE_right (s)	5.29 (±6.95)	10.30 (±8.27)	−1.09*
	Single-leg stance _CE_left (s)	3.74 (±5.06)	10.53 (±10.14)	−3.27*
	Functional reach test _right (cm)	32.55 (±10.97)	38.34 (±7.82)	−1.60
	Functional reach test _left (cm)	30.96 (±10.06)	40.21 (10.61)	−2.34*
Aerobic Condition	Two-minute step test _before (bpm)	90.29 (±12.81)	83.69 (±21.70)	1.91
	Two-minute step test _after (bpm)	112.63 (±19.50)	105.31 (±19.19)	0.35
	Two-minute step test _2min after (bpm)	94.21 (±12.91)	88.25 (±20.75)	1.39

*OE* opened eyes, *CE*: closed eyes

*: *p* < 0.05

Some physical tests such as hand strength (HGT) and balance tests (SLS and FRT) demonstrated that the group of active people had better performance than the group of inactive people. In contrast, As for the physical test that assessed flexibility and aerobic condition, significant differences were not found with the group of people physically inactive in spite of the group of active people had better performance in all assessed tests in flexibility, strength, balances and aerobic condition.

As for the physical test that assessed flexibility and aerobic condition, even though the group of active people had better performance in these tests, significant differences were not found with the group of people physically inactive.

## Discussion

The present study explores the physical fitness of people with SMI and makes comparisons in relation to their levels of physical activity, To our knowledge, this study is the first to show the differences between the two groups of different levels of physical activity in people with SMI, and finds that there were significant differences in both the balance and the hand strength of people with SMI.

It is important to highlight the better performance in muscle strength and balance tests found in the group of physically active people, as these two variables (strength and balance) have been described as important predictors of serious public health problems, like risk of falls or sarcopenia [38, 39]. Furthermore, it should be noted that physical activity in people with SMI is an important indicating factor for these people to have a better quality of life and be independent in carrying out the activities of daily life for as long as possible [8, 9, 40]. Moreover the improvement in physical fitness is one of the main reasons to engage in physical activity in this population [41].

Physical fitness in people with SMI has been measured in other studies [42, 43] with a different battery of physical tests, so the results of this study in terms of the physical tests used cannot be compared with similar studies. However, the study of Vancampfort et al. 2016 [43] in the Eurofit battery test used three physical tests similar to those used in the present study. Firstly, the SLS test with open eyes (physically active people 16.20 ± 11.79; inactive people 12.47 ± 10.69) found similar results to the study of Vancampfort et al., which used the Flamingo balance test (16.75 ± 9.00). Secondly, the HGT (physically active people 28.00 ± 12.33; inactive people 26.11 ± 11.91) obtained better performance in this test in Vancampfort et al. with a value of 40.55 ± 11.75. While for the abdominal strength test, the values of the present study were higher than those found in the study of Vancampfort et al., with a value of 15.11 ± 7.60 for physically active people and 15.26 ± 10.80 for inactive people against a value of 10.50 ± 8.00 in the study of Vancampfort et al. Despite the differences in some physical tests between the present study and the study of Vancampfort et al., it is important to highlight that the participants were similar in terms of age (46.21 ± 8.37 present study; 40.55 ± 10.1 study of Vancampfort et al.); whereas, the present study included a higher number of diseases listed as SMI, while the study of Vancampfort et al. included only people diagnosed with bipolar disorder and schizophrenia.

The differences in physical fitness between active and inactive people with severe mental illness have only been shown in the Vancampfort et al. study and in the present study, although these differences have been shown in another population as intellectual disability [44], Although in many studies have shown the benefits of physical activity in improving physical fitness in this population [45, 46].

The present study demonstrates the difference in physical fitness between people with SMI who practise physical activity and those who do not practise physical activity, showing better performance of physical fitness in physically active people. Different physical activity interventions were carried out in which there was shown an improved physical fitness in people with SMI [11, 47], however, these interventions should not just focus on aerobic activity but also on increasing the muscle strength and balance of people with SMI and also encourage these people to perform physical activity independently and to decrease the risk of falls similar to those carried out in other populations as elderly people [48].

This better values in physical fitness shown in the present study into both strength and balance suggests that physically active people with SMI also experience a lower risk of falls and fractures, which are common due to high doses of anti-psychosis drugs which cause a loss of balance in this population [49].

## Conclusions

In conclusion, the present study is the first to show differences in physical fitness in people with SMI that practise physical activity and those who do not practise physical activity, showing a significant difference in both muscle strength and balance between both groups, with better performance found for physically active people; although the physical fitness of people with severe mental illness is not only identified by their level of physical activity, we must focus on the variables that improve the physical fitness of these people to carry out future interventions.

## Limitations

The present study has a number of limitations. Due to the large number of diseases that are included as SMI, it was not possible to find enough people with the same disease to conduct the study. Additionally, we did not use a standardized physical activity assessment and future efforts should include assessments with established reliability. Finally, being a cross-sectional study, it was not possible to affirm a causal relationship. On the other hand, the present study evaluate the physical fitness in people with SMI according their level of physical activity and the large number of assessed physical tests allows us to determine the physical fitness of this population across a large set of variables but in the future the present results could be improve finding the differences between different pathologies included in severe mental illness.
